# Using Machine Learning to Understand the Causes of Quantum Decoherence in Solution-Phase Bond-Breaking Reactions

**DOI:** 10.1021/acs.jpclett.3c03474

**Published:** 2024-01-19

**Authors:** Kenneth J. Mei, William R. Borrelli, Andy Vong, Benjamin J. Schwartz

**Affiliations:** Department of Chemistry & Biochemistry, University of California, Los Angeles, Los Angeles, California 90095-1569, United States

## Abstract

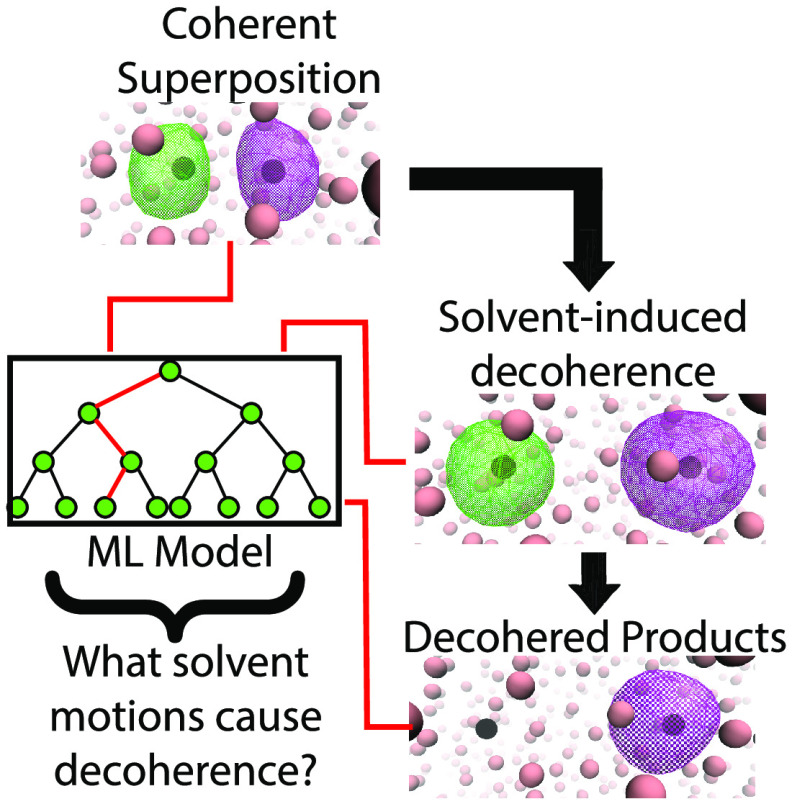

Decoherence is a fundamental phenomenon that occurs when an entangled quantum state interacts with its environment, leading to collapse of the wave function. The inevitability of decoherence provides one of the most intrinsic limits of quantum computing. However, there has been little study of the precise chemical motions from the environment that cause decoherence. Here, we use quantum molecular dynamics simulations to explore the photodissociation of Na_2_^+^ in liquid Ar, in which solvent fluctuations induce decoherence and thus determine the products of chemical bond breaking. We use machine learning to characterize the solute–solvent environment as a high-dimensional feature space that allows us to predict when and onto which photofragment the bonding electron will localize. We find that reaching a requisite photofragment separation and experiencing out-of-phase solvent collisions underlie decoherence during chemical bond breaking. Our work highlights the utility of machine learning for interpreting complex solution-phase chemical processes as well as identifies the molecular underpinnings of decoherence.

The fact that quantum systems can exist in a superposition of coherent quantum states is what gives rise to their utility in the emergent field of quantum information science. When such an entangled quantum system interacts with a fluctuating environment, motions of the bath can make a “measurement” on the system, breaking the entanglement and collapsing the system into an eigenstate.^1−5^ This phenomenon, known as quantum decoherence, provides the key limitation on technologies such as quantum computing, quantum communications, and quantum metrology.^6−8^ The usual approach to decreasing the rate of quantum decoherence is simply to lower the temperature, thus reducing the frequency and amplitude of bath fluctuations that couple to the entangled quantum system.^6^

Despite all the interest, there are only a handful of studies^9−19^ that have worked to provide a microscopic picture of how bath motions couple to a quantum system and cause decoherence or that investigate whether restricting certain types of bath motions might allow chemical systems to remain entangled at higher temperatures. Most common theoretical approaches are derived from a generalized master equation and treat the loss of quantum coherence by introducing empirical off-diagonal terms in the system density matrix,^2,5,20−22^ which provides little insight into understanding precisely what types of underlying bath motions or coupling are responsible. A few studies have examined decoherence using an explicit bath representation, notably the works of Sanz et al.^23^ and Elran et al.,^24^ who used a classical analogue approach involving a Wigner distribution for initial quantum states and molecular dynamics simulations to study the vibrational decoherence of I_2_ in a bath of liquid xenon.

In this work, we use quantum molecular dynamics simulations to examine the quantum decoherence that accompanies the breaking of chemical bonds in solution. The decoherence event we study is the solvent-induced collapse of a bonding electron’s wave function. This wave function is initially prepared by photoexcitation in a superposition of positional states, with the electron residing equally on both possible photofragments. After decoherence, the electron localizes onto a single positional state associated with only one of the two photofragments, determining the products of the bond-breaking reaction.

For simple molecules that involve one-electron bonds, such as the Na_2_^+^ molecule considered here, the wave function of the bonding electron is described as a coherent superposition of quantum states centered on each atom, analogous to a superposition of quantum spin states.^25^ In a vacuum, this coherence is conserved indefinitely, even as the bond length approaches infinity; in other words, half the bonding electron remains on each atom as the bond is broken. In the condensed phase, however, interactions of the quantum system and the solution environment break the local symmetry, causing decoherence via collapse of the wave function onto a single positional quantum state. In other words, decoherence determines whether dissociation of molecules like Na_2_^+^ produces Na + Na^+^ or Na^+^ + Na, so that understanding the motions that cause decoherence is highly chemically relevant.

To study interactions of the bonding electron with a solvent environment, we focused our simulation efforts on the excited-state dissociation of Na_2_^+^ in liquid Ar. This particular molecular system is well understood in the gas phase and has been simulated in solvated clusters by Douady et al.^26^ In our previous work on this system in liquid Ar, we found that the solvation response during dissociation deviates significantly from linear response predictions and that the system experiences discrete solvent environments as the molecule’s bond lengthens.^27,28^

Here, we take advantage of machine learning (ML) methods to focus on the detailed molecular motions of the liquid Ar bath underlying quantum decoherence and wave function collapse. Although ML is conventionally used as a means to extend the system size and/or time scales in quantum simulations, here we use it to determine which part of a high-dimensional feature space, in this case the solvent motions, can predict decoherence. Using a balanced random forest (BRF) classifier model, we show that we can identify the solvent motions that cause decoherence with ∼79% accuracy, given an optimized feature space with only five dimensions. The results of our feature importance analysis indicate that there are two primary requisites for decoherence. First, decoherence is induced by asymmetric collisions where solvent atoms strongly interact with one Na^+^ but not the other. Second, decoherence cannot occur until the dissociating molecule reaches longer bond distances, suggesting a transition from a single molecular entity experiencing unified solvent collisions to separate photofragments undergoing independent local solvent fluctuations.

Our simulations use mixed quantum/classical (MQC) molecular dynamics (MD) simulations, where the bonding electron is described quantum mechanically and the solvent motions are described classically. Interactions between the bonding electron and classical particles are treated through previously developed pseudopotentials.^29−31^ The details of the methods are the same as those used in our previous work^27,28,32−37^ and can also be found in the Supporting Information. Briefly, the system is composed of a single Na_2_^+^ solute and 1600 Ar atoms. We take 210 uncorrelated, ground-state, equilibrium configurations and launch nonequilibrium trajectories by promoting the bonding electron in these configurations onto its first excited state. The dynamics are propagated nonadiabatically using Tully’s fewest-switches surface hopping algorithm, although none of the trajectories underwent a surface hop to the ground state prior to the decoherence event of interest. The nonequilibrium dynamics were followed for 2 ps, a time sufficient to see decoherence in the majority (91.4%) of trajectories.

It is worth noting that the word “decoherence” does not have a single precise meaning in the literature. For example, coherence between adiabatic electronic states induced by motions of an external bath is frequently investigated in surface hopping studies,^38^ and the subsequent transitions between states (“surface hops”) are often termed decoherence events.^39^ Rather than the mixing of electronic states induced through the nuclear degrees of freedom, however, in this work, we use the word decoherence to refer to charge localization events that take place on a single adiabatic electronic state. As described further below, we choose to think of the single bonding electron on the lowest adiabatic excited state of Na_2_^+^ as being in an entangled/coherent superposition of Na^+^ + Na^0^ and Na^0^ + Na^+^ states. Here, we define the decoherence event as the solvent-induced suppression of interference between these positional quantum states of the bonding electron, which causes localization of the electron onto a single Na. As mentioned above, this event generally takes place well before any instances of surface hopping onto the adiabatic ground electronic state. A similar definition of decoherence has been used in studies of molecular shape through localization of nuclei by Mátyus and Cassem-Chenaï.^40^

We begin our exploration of the decoherence that occurs following the photodissociation of Na_2_^+^ in liquid Ar by examining the basic features of this chemical process. Figure 1a shows snapshots from a representative nonequilibrium photodissociation trajectory, which begins with the molecule in its electronic ground state (upper left panel). At time zero, we promote the molecule to its lowest electronic excited state (upper right panel), introducing a node in the bonding electron wave function. This is a classic σ to σ* transition, where the lack of excited-state electron density between the two nuclei initiates the bond-breaking process.

**Figure 1:**
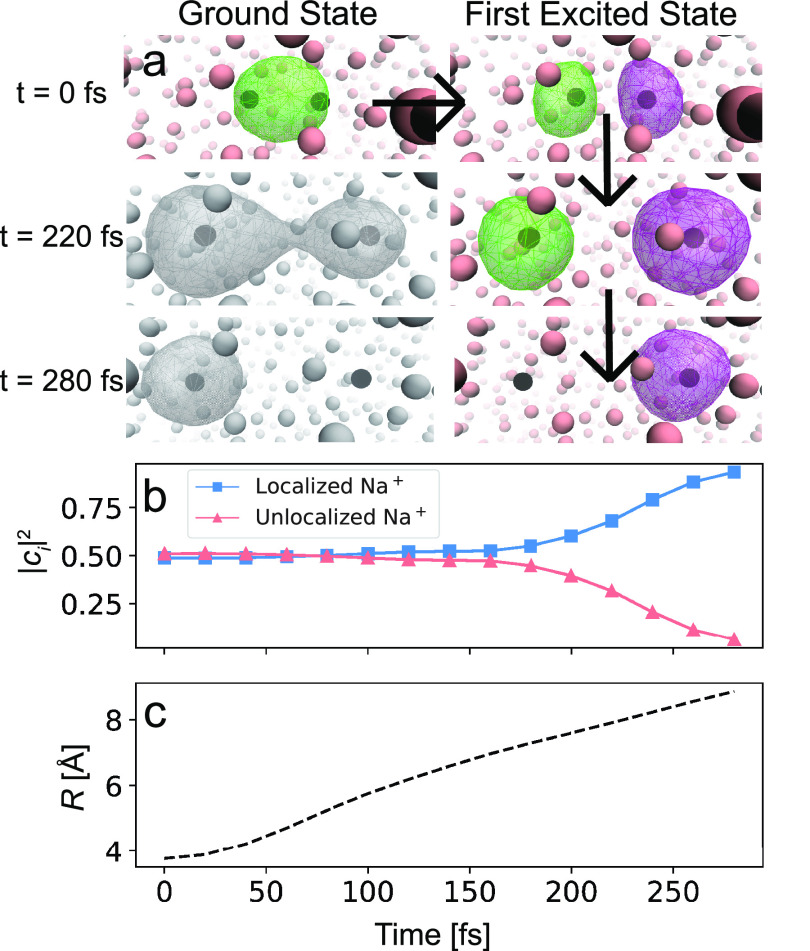
Analysis of a single nonequilibrium trajectory of the photodissociation and subsequent decoherence event of excited Na_2_^+^ in liquid Ar. Panel a shows snapshots during the dissociation process. The black spheres represent the Na^+^ cores, the pink spheres correspond to argon atoms, and the wire mesh depicts the wave function of the bonding electron. Each trajectory is initiated from an equilibrium ground-state configuration, and at time zero (top left), the electron is promoted to its first excited state, introducing a node in the wave function with about equal amplitude on each Na^+^ (top right). As the bond distance (*R*) elongates, solvent fluctuations introduce asymmetrical environments around each photofragment. This causes the wave function amplitude to start to move onto a single Na^+^ (middle right) by 220 fs, the beginning of quantum decoherence. By 280 fs, the wave function is essentially fully localized (≥90% onto a single Na^+^ (bottom right)), and the decoherence event is complete. Snapshots in gray (middle and lower left) depict how the unoccupied ground-state wave function evolves during the nonequilibrium excited-state trajectory. Panel b tracks the squared amplitude of the individual Na^+^ quantum states that comprise the coherent superposition. Panel c shows the time history of the Na^+^–Na^+^ distance for this trajectory, which reaches a separation of ∼9 Å at the time of localization.

The dissociative σ* state can be described as a coherent superposition of localized states where the electron is associated either entirely with the left Na^+^, which we denote |Na_(1)_⟩, or entirely with the right Na^+^, which we denote |Na_(2)_⟩. Thus, at the instant of Franck–Condon excitation, the one-electron wave function takes the form
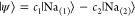
1where *c*_*i*_ are the amplitudes of the individual atomic quantum states, the minus sign indicates that the two localized states have opposite phase, and |ψ⟩ is the total wave function of the excited bonding electron.

Following the initial excitation, the Na_2_^+^ molecule begins to dissociate. In the gas phase, the *c*_*i*_ coefficients describing the wave function of the dissociating molecule are equal (with both |*c*_*i*_|^2^ = 0.5), and they remain so as the dissociation proceeds because there is no environment to break the symmetry; a movie of this process based on a gas-phase simulation trajectory is available in the Supporting Information. In the condensed phase, the interaction of each dissociating Na^+^ with its local solvent environment alters the coefficients comprising the total wave function. The center-right panel in Figure 1a shows that 220 fs after photoexcitation, the wave function starts becoming asymmetric, with a larger amplitude on the right-hand Na^+^. By 280 fs (bottom right panel in Figure 1a), the wave function localizes on the Na^+^ on the right. A movie of a typical condensed-phase trajectory is also available in Supporting Information.

Figure 1b plots the time-dependent coefficients that describe the total wave function for this trajectory, with |*c*_1_|^2^ shown as the pink triangles and |*c*_2_|^2^ shown as the blue squares. As suggested in Figure 1a, the coefficients start off equal, but over a relatively short time scale between 220 and 280 fs, one of the coefficients rapidly goes to zero, while the other approaches unity, the hallmark of a quantum decoherence event. Figure 1c shows the distance between the two Na nuclei as a function of time for this trajectory, which starts at the Na_2_^+^ equilibrium bond length of 3.9 Å. The inflection point seen near ∼100 fs represents a strong collision of the dissociating fragments with the surrounding solvent cage,^27,41^ but this relatively violent molecular event is clearly not what is responsible for decoherence, which does not start to for another ∼80 fs. The goal of this study is to determine what solvent configurations or motions cause quantum decoherence in the condensed phase.

To this end, we start by examining our nonequilibrium ensemble of 210 trajectories simulating the dissociation of Na_2_^+^ in liquid Ar to examine the variety of conditions under which decoherence occurs. For the purposes of this paper, we define decoherence as taking place when one of the |*c*_*i*_|^2^ values is ≥0.9. In the left inset of Figure 2, we have plotted the distribution of times when decoherence events occur. The most probable time for decoherence to take place is ∼260 fs after excitation, but the distribution has a long tail reflecting the fact that a significant number of trajectories take a very long time for decoherence to occur. The right inset in Figure 2 shows the distribution of Na–Na bond distances at the moment of decoherence; decoherence clearly never occurs unless the dissociating bond length has reached at least 8 Å. This suggests that decoherence cannot take the reaction to completion until the bond is significantly longer than that in its ground-state equilibrium.

**Figure 2:**
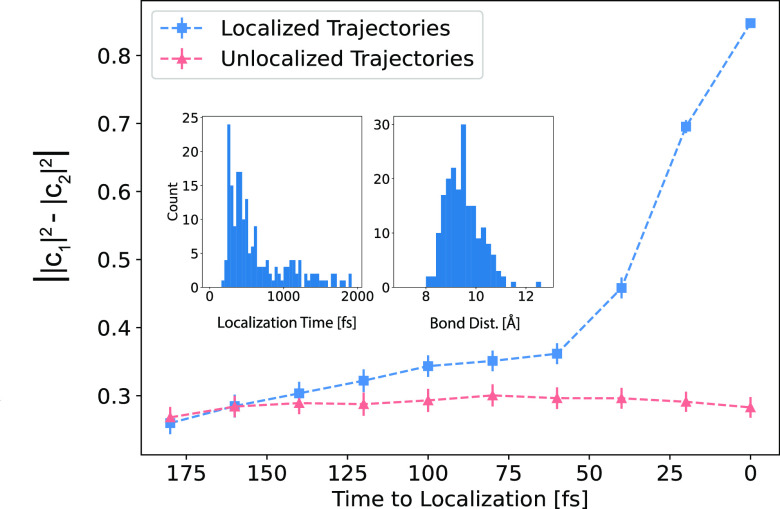
Nonequilibrium ensemble average of the absolute difference between the |*c*_*i*_|^2^. The blue curve shows the coefficient difference in |*c*_*i*_|^2^ where time zero at the right of the *x*-axis is the moment at which the decoherence is complete. Clearly, the localization event is not instantaneous but happens over a time scale of ∼60 fs, starting when the slope of the difference between the |*c*_*i*_|^2^ dramatically increases. The pink curve is the difference in |*c*_*i*_|^2^ over 180 fs windows in the 8.4% of the trajectories where electron localization does not occur, serving as a baseline for understanding the decoherence events. The insets show the distribution of times (left) and bond distances (right) at which localization occurs. Although the most probable localization time is ∼260 fs, some trajectories take much longer for decoherence to occur. The bond distance distribution when localization occurs suggests that having a photofragment separation of at least ∼8 Å is a prerequisite for quantum decoherence in this system.

Because the decoherence events happen over a broad range of times between trajectories, in Figure 2 we examine the nonequilibrium ensemble average behavior of the wave function coefficients over the 180 fs time window immediately prior to localization. Here, time zero is the time of the decoherence event in each trajectory, and the blue squares show the absolute difference in the |*c*_*i*_|^2^ coefficients in the time preceding and up to the decoherence event. As a control, the pink triangles show the same quantity averaging over 180 fs windows of the 8.4% of nonequilibrium trajectories for which electron localization does not occur. The coefficient differences in trajectories for which decoherence occurs and those where the system remains in a coherent superposition begin at the same value but then diverge from each other starting about 60 fs before the decoherence event. This indicates that decoherence on the excited state of Na_2_^+^ is not instantaneous but instead depends on some particular solute–solvent interaction that occurs on an ∼60 fs time scale.

Due to the atomic nature of the Ar bath, decoherence must be caused by translational motions of the solvent. However, it is unclear whether there is a single solvent interaction that causes quantum decoherence or a collective event that can only occur under specific conditions. To find out what solvent motions break the symmetry of the photoexcited molecule and cause decoherence, we examined a number of order parameters that encode solvent atomic positions, atomic velocities, solute–solvent forces, and components of the solute–solvent interaction energies; descriptions of some of the parameters we explored are given in the Supporting Information. Unfortunately, no single parameter that we calculated was sufficient to completely describe the observed decoherence events. What we show next, however, is that using combinations of these parameters as a high-dimensional feature space for a ML model, we were able to make effective predictions for when decoherence occurs.

Our approach is to cast the decoherence event as a classification problem, where we seek to predict whether the electron will localize on Na_(1)_ (class 1), localize on Na_(2)_ (class 2), or remain delocalized (class 3). From each of our nonequilibrium trajectories where localization occurred, we took the last 9 time steps prior to the decoherence event, yielding 1890 examples from which to train and test the model. To encode the local environment of each Na^+^, we calculated numerous features including atom-centered symmetry functions^42^ describing pairwise solvent distances and angles, solute–solvent forces, solute–solvent velocities, and various components of electron–solvent energies, to name a few. After much investigation, described in more detail in the Supporting Information, we found that only five features were needed to give both sufficient accuracy and relatively low dimensionality for interpretability. The feature set includes the dimer bond length, the integrated solvent potential felt by the electron around each Na^+^ (integrated over a radius of 2.6 Å), and whether or not each Na^+^ experiences a collision with an Ar solvent atom (determined through changes in Na^+^ velocity angles). The details of our feature engineering are further discussed in the Methods section as well as in the Supporting Information.

One issue with building our ML model is that each trajectory in our ensemble has only one decoherence/localization event, so that our data are highly imbalanced toward the unlocalized class. To handle this imbalance without severely reducing the size of our training and test sets through downsampling, we used a BRF classifier,^43,44^ which implements undersampling for each bootstrap sample to reduce bias in model training. Model performance was validated using a balanced accuracy score,^45^ which scales the normal prediction accuracy by class-balanced sample weights. The optimized model achieved a single train/test split balanced accuracy score of ∼79% and a cross-validated balanced accuracy of ∼78%.

To interpret the resulting model and draw insights about the underlying causes of condensed-phase quantum decoherence, we employed a SHapley Additive exPlanations (SHAP) analysis.^46,47^ SHAP values quantify the impact of each feature on the final prediction of a model. In short, a SHAP analysis takes a coalition (a subset of the features) and calculates the marginal contribution of adding that feature compared to leaving it out. The prediction probability for a class is the sum of all of the feature SHAP values along with the expected model output. For our trained BRF, the expected (random) probability for each of the three classes is 33.3%. Thus, positive SHAP values for a feature enhance prediction of that class, while negative SHAP values reduce prediction of that class. Figure 3 summarizes the results of the SHAP analysis for the three most important features, and the full SHAP analysis is available in the Supporting Information.

**Figure 3:**
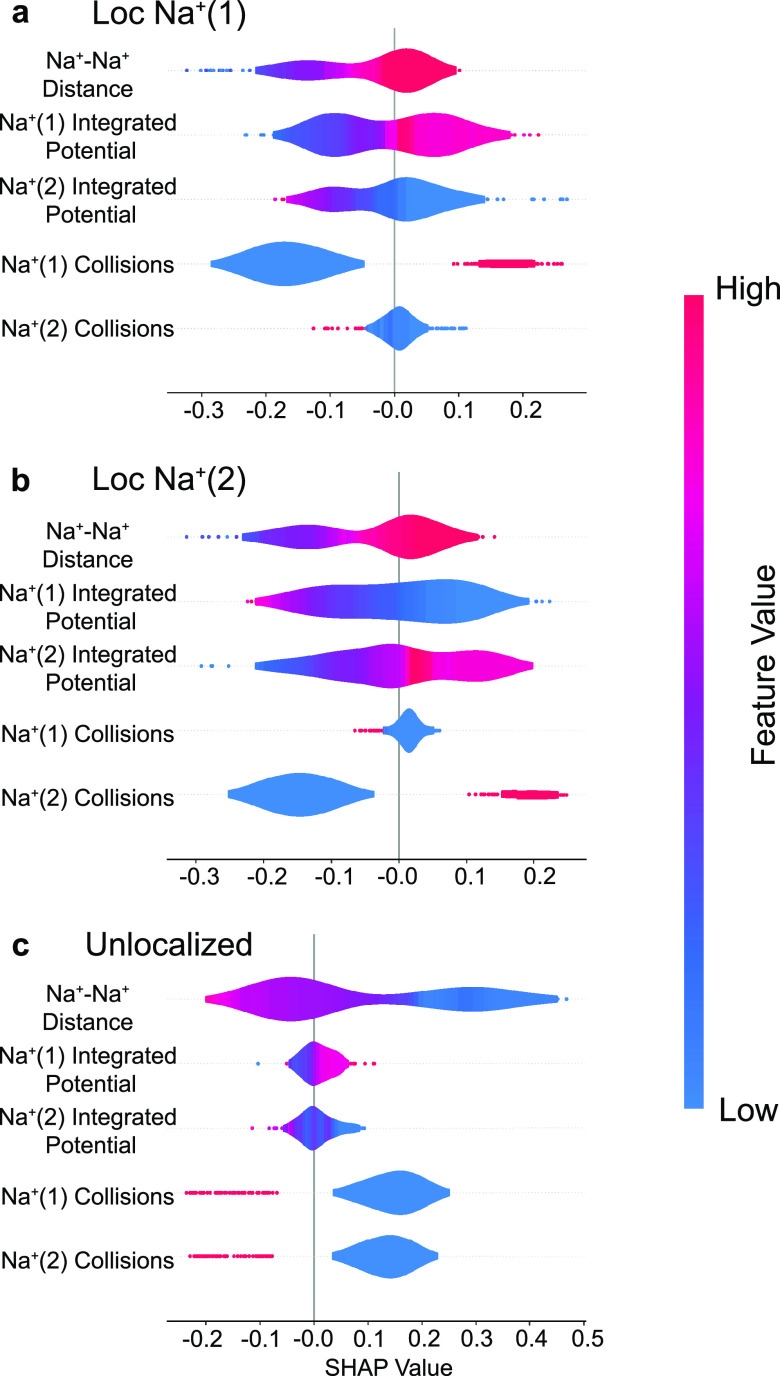
SHAP analysis on the BRF classifier model for predicting the role of different features on three classes of events: decoherence with electron localization on Na_(1)_^+^ (panel a), decoherence with electron localization on Na_(2)_^+^ (panel b), and the system remaining in a coherent superposition with the electron delocalized between the two Na^+^’s (unlocalized, panel c). The color scale represents the value of each feature. The SHAP value is calculated for each feature and represents the deviation of each class from the random output of 1/3. Negative SHAP values decrease the likelihood of this class prediction, and positive SHAP values increase the likelihood for the model to predict this class based on that feature contribution. SHAP values near zero do not impact the prediction. For the localized on Na_(1)_^+^ class prediction (panel a), large bond distances, higher collisions of Ar solvent atoms with Na_(1)_^+^, and low collisions of Ar with Na_(2)_^+^ create a positive likelihood for the BRF model to predict decoherence via electron localization onto Na_(1)_^+^. Panel b shows that the localization on Na_(2)_^+^ class behaves similarly, with positive correlations for large bond distances and collisions only on Na_(2)_^+^. Thus, the key requirement for decoherence is the presence of an asymmetric collision (Figure 4), with the electron localizing on the photofragment that experiences the collision. The SHAP analysis in panel c shows that small bond distances and either a lack of collisions or simultaneous collisions on both Na^+^’s increase the likelihood of the model to predict the unlocalized class.

Figures 3a and 3b show the SHAP distributions for predicting decoherence via electron localization on Na_(1)_ or Na_(2)_, respectively. For both of these class predictions, large Na–Na bond distances are associated with electron localization and quantum decoherence, whereas shorter bond distances maintain coherence and promote electron delocalization, as seen in panel c. This agrees well with the decoherence bond length distribution shown in the inset of Figure 2. Perhaps most strikingly, however, collisions between the Ar solvent atoms and the different Na^+^’s show the strongest effect for predicting decoherence and electron localization, with no feature attributions seen near zero. Collisions with a particular Na atom are strongly correlated with electron localization onto that Na atom, while a simultaneous collision with the other Na atom is anticorrelated with decoherence, as can be seen in the negative tail of the SHAP values, showing high feature values. Moreover, for the unlocalized class predictions shown in panel c, we see that a lack of collisions on either Na enhances delocalization. All of this indicates that solvent collisions that occur with one Na atom but not the other are a necessary condition for quantum decoherence.

As mentioned above, the electron prefers to localize on the Na^+^ that experiences the collision. Although seemingly counterintuitive, this is because the dissociation takes place adiabatically on the excited state^28^ and the electron preferentially localizes on the higher-energy photofragment, a phenomenon known in the literature as “anomalous charge flow”.^48^ Perhaps of even more interest is the fact that most collisions do not lead to quantum decoherence. One way to visualize the presence of solvent collisions with the photofragments is by plotting the change in the angle of the velocity vector of each Na^+^ (cos^–1^[*v̂*_Na_(*t*)·*v̂*_Na_(*t* + δ*t*)], where we choose δ*t* = 20 fs), which we refer to as the collision angle, as shown in Figure 4a for the same representative trajectory explored in Figure 1. In Figure 4a, the blue curve corresponds to the collision angle for the Na^+^ onto which the electron eventually localizes, while the pink curve shows the collision angle for the other Na^+^. We identify collisions as occurring when the collision angle shows a maximum, reflecting that the Na^+^ velocity vector significantly changed the angle due to large local forces from interactions with the Ar solvent.

**Figure 4:**
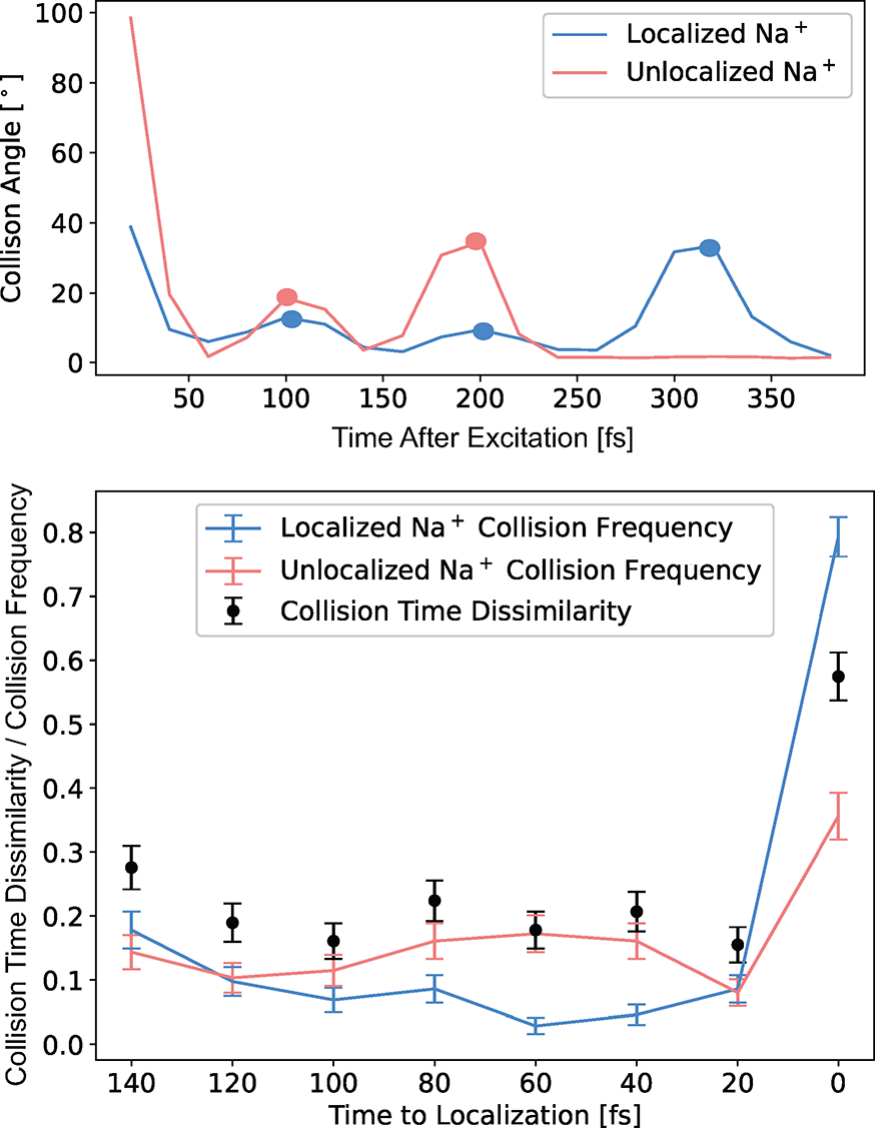
(a) Collision angles, calculated as the angle between the instantaneous Na^+^ velocity at time *t* and at time *t* – 20 fs at each time step, for the Na^+^ onto which the electron eventually localizes (blue curve) and the other Na^+^ (pink curve) for the same representative trajectory explored in Figure 1. There are three strong solvent collisions with the Na^+^ onto which the electron localizes at ∼100, 200, and 320 fs, but only two collisions, at ∼100 and 200 fs, with the other Na^+^. In this example, localization occurs at 280 fs, and the collision angles show a peak just after the localization time on the localized Na^+^ and the absence of a peak on the unlocalized Na^+^. The inset shows an illustration of the binary representation of the collision vector (*v⃗*_*i*_) used to calculate the collision time dissimilarity. (b) Nonequilibrium ensemble average of the differences in collision times for each Na^+^. We calculate the collision time dissimilarity (black dots) as the ensemble-averaged absolute difference in collision vectors, which represents the degree of dissimilarity in the collision times between each dissociating Na^+^. On average, the collision phases for the two Na^+^’s are quite similar from 140 to 20 fs before localization, but at the moment of localization the degree of collision dissimilarity sharply increases. The pink and blue points, connected by lines to guide the eye, show the frequency of collisions on the Na^+^ onto which the electron eventually localizes (blue) and the other Na^+^ (pink). Prior to localization, we see that collisions do occur, but the collision times between the two Na^+^ are similar. At the time of localization, the collision frequency is much higher for the Na^+^ onto which the electron localizes, and the high dissimilarity value shows that during localization collisions do not occur simultaneously on both Na^+^’s.

Figure 4a shows that the first collision on each Na^+^ occurs at ∼100 fs; this is the so-called caging event,^27,49^ where the dissociative force drives the photofragments strongly into the first-shell solvent atoms. This event, although relatively violent on a molecular scale, does not induce decoherence both because it happens effectively simultaneously for the two Na^+^’s and because the system has not yet reached the requisite ∼8 Å bond distance. In other words, at small bond distances, both photofragments are effectively coupled to a single bath, maintaining coherence, even in the presence of strong solvent collisions. Once the fragments reach a sufficient separation, each effectively experiences a separate local bath, allowing collisions to alter the degree of coherence. For the example in Figure 4a, the bond length is near the 8 Å requirement for separate local environments at the time of the second set of collisions, ∼200 fs, but decoherence is not induced because the collisions occur essentially simultaneously, maintaining the two-fragment entanglement. It is not until the onset of the third sharp collision, peaking at 310 fs, which occurs only with a single Na^+^, that the wave function localizes and decoherence takes place. These findings fit well with the SHAP analysis in Figure 3, where high values for the localized Na^+^ collision vector and low values for the delocalized Na^+^ collision vector increase the likelihood of predicting the localized class.

To further explore the correlation between asynchronous collisions and quantum decoherence, we have developed a parameter to quantify the degree of dissimilarity in collision times between the Na^+^’s for the nonequilibrium ensemble. The parameter is based on a binary representation of the collision angles shown in the inset of Figure 4a, the same feature used in our ML analysis. We define the peaks of the collision angles on a given Na^+^ as “1” and the rest of the time points as “0”, creating a vector of collision events over time. We then take as our metric the absolute difference between these binary vectors for each Na^+^ as our collision dissimilarity parameter, averaging over the nonequilibrium ensemble to generate the black dots plotted in Figure 4b. By this measure, the degree of dissimilarity between collision times prior to electron localization is relatively low, but at the moment of the decoherence event, the degree of dissimilarity sharply increases. Because we define this measure as a binary vector, the only way it can be nonzero is when there is a collision on one Na^+^ but not the other. Superimposed in Figure 4b is the collision frequency for the Na^+^ onto which the electron eventually localizes (blue data points/lines) and for the other Na^+^ (pink data points/lines). The data show that the Na^+^ onto which the electron localizes experiences a collision at the moment of localization ∼80% of the time. As described in the Supporting Information, we also considered features such as the local solvent density (Figure S2), the collective solvent velocities (Figure S3), and the absolute difference in the solvent potential between each Na^+^ (Figure S1), the behavior of all of which is consistent with the idea that asynchronous solute–solvent collisions are what induces quantum decoherence.

This idea of asynchronous solute–solvent collisions coinciding with decoherence makes sense with our understanding of quantum systems. If two positional quantum states of a system are highly entangled, as when the bond length is short, then the interactions “local” to one site also impact the wave function situated on the other site. Moreover, simultaneous collisions do not cause decoherence, even when the bond length is sufficiently long. This would suggest that experiencing collisions is not necessarily detrimental to preserving quantum coherence, as long as the interactions and timing on each fragment are not too different. Thus, rather than simply trying to minimize collisions and interactions with the environment, our results suggest that coherence could be preserved if one could design the quantum system in such a way that the collisions would act symmetrically on the entangled particles. One also can design the system to maintain entanglement,^50^ such as is the case for our system when the bond length is less than 8 Å, where the electron experiences only a single set of fluctuations that cannot induce decoherence even if the interactions with the environment are strong.

In summary, we have explored the microscopic mechanisms underlying quantum decoherence during a simple chemical reaction, the photodissociation of Na_2_^+^ in liquid Ar. We found that with the aid of machine learning we were able to provide a molecular interpretation of the chemical events underlying quantum decoherence and electron localization in this system, which is what determines the products of this simple reaction. The use of machine learning turned out to be critical to our analysis because the microscopic bath motions underlying decoherence could not be reduced to a singular molecular event; instead, our ML model suggests that decoherence requires a higher-dimensional description. For the photodissociation of Na_2_^+^ in liquid Ar, the primary environmental factors that influence decoherence are a requisite spatial separation of the entangled positional atomic states as well as a need for asynchronous solute–solvent collisions on each photofragment. Thus, the time evolution of entangled positional quantum states is determined by collective motions of the bath rather than any specific single interaction.

We close by noting that decoherence of quantum states in condensed-phase systems is not limited to the bond breaking of diatomic molecules but is fundamental throughout chemistry^51−53^ as well as present in biological systems^54−58^ and has direct applications to emergent fields such as quantum computing, sensing, and communications.^59,60^ The conclusions we have drawn in this work, particularly the requirement for decoherence resulting from dissimilar interactions with the entangled quantum particles, should extend generally to coherent quantum systems.

## Methods

### Overview of Simulation Details

In our MQC MD simulations, the Na^+^ cores and the argon solvent atoms are treated classically while the single bonding electron of Na_2_^+^ is treated quantum mechanically, giving us the respective classical and quantum subsystems. The classical subsystem is treated as a Lennard-Jones (LJ) fluid with pairwise LJ interactions between particles. The quantum subsystem consisting of the single bonding electron is treated using a 32^3^ grid basis set within our simulation box. The time-independent Schrödinger equation is solved for our quantum subsystem at every time step. Interactions between the classical and quantum subsystems are accounted for using Phillips–Kleinman^61^ (PK) pseudopotentials that have been previously developed and benchmarked.^29,30^ Contributions by the quantum subsystem to the classical nuclear dynamics are calculated through the Hellman–Feynman force.

The simulation box contains two Na^+^ cores to model our solute and 1600 argon atoms to model the bulk solvent. The box length is set to 43.8 Å, and the quantum subsystem grid spans a length of ∼25 Å centered at the origin of our simulation box. A time step of 4 fs was used with the velocity-Verlet algorithm to propagate the classical particles. All simulations were performed in the (*N*, *V*, *E*) ensemble at a temperature of 120 K. The work presented here is from a series of 210 nonequilibrium trajectories of the photodissociation of Na_2_^+^ in liquid argon. The initial configurations for each individual trajectory were taken from uncorrelated time steps of a ground-state equilibrium simulation of Na_2_^+^ in liquid argon. The bonding electron in each trajectory is promoted to its first excited state, and the dynamics are allowed to propagate for 2 ps in order to study the early time dynamics on the first excited state. Nonadiabatic transitions are enabled by using the FSSH algorithm. Further discussion on all simulation details can be seen in the Supporting Information.

### Collision Angle Dissimilarity

The collision angles for each Na^+^ are calculated by using their instantaneous velocities. At each time step, the angle is calculated between the Na^+^ instantaneous velocity vector at time *t* and the instantaneous velocity vector for that same Na^+^ at time *t* – 20 fs. A peak finding algorithm in Mathematica^62^ was used to detect the collision times for each Na^+^ within each trajectory for our entire ensemble.

The collision time dissimilarity is calculated by first expressing the collision angles for each Na^+^ as a binary vector. The length of the vector is equal to the number of time steps in the trajectory, and the value at each time step is 0 if there are no collision peaks detected and 1 if there is. For example, the localized Na^+^ in the example trajectory plotted in Figure 4 has a binary vector with 3 instances of 1 and the rest 0 in that time regime. We then take the difference between the localized and unlocalized Na^+^ binary vector at each time step, where a difference value of zero indicates no difference in the collisions at that time step and a difference value of one indicates a collision on one Na^+^ but not the other. The difference between Na^+^ binary vectors is ensemble averaged in the 140 fs time window prior to localization.

### Machine Learning Analysis

#### Feature Engineering and Selection

The features used in training the BRF model included the dimer bond distance, the effective volume around each sodium, a spherically integrated pseudopotential value around each sodium, solvent atom-centered symmetry functions for each sodium core, and binary sodium collision vectors. We trained models on all permutations of features and chose the smallest subset that produced the best balanced accuracy results on a validation set. This final feature set included the dimer bond distance, the integrated Na^+^ pseudopotential, and binary Na^+^ collision vectors, giving a dimensionality of five. For further information on the feature set, feature selection, and hyperparameter optimization of the atom-centered symmetry functions as well as the BRF model, see the Supporting Information. Before model training and testing, all input data except for the binary collision vectors were standardized.

#### Balanced Random Forest Training Performance Validation

We trained and evaluated both a balanced random forest classifier and a balanced bagging classifier. Over all performance metrics, including replicate test/train splits, single test/train splits, and *k* = 5 cross-fold validation, the balanced random forest model performed better than the balanced bagging model. *K*-fold cross-validation used all 1890 data points, while 80/20 train/test splits were used for test/train split validation. All models were implemented in Python 3.9.4 using the imblearn 0.7.0 package,^44^ and evaluated using scikit-learn 0.24.2.^63^ Because every 9 data points in our data set came from correlated trajectories in our ensemble, a time-series data split was done to avoid data leakage that would artificially boost model performance. That is certain trajectories were assigned to the training data, while completely separate trajectories were assigned to the test data. As indicated above, our BRF model achieved a balanced accuracy score of 78%. A learning curve of the model can be seen in the Supporting Information.

#### SHAP Analysis

SHAP values were calculated using the Python-implemented version 0.41.0.^46^ Beyond the violin summary plots shown above, SHAP feature importance plots also can be found in the Supporting Information.

## Data Availability

The computer code used in this study as well as any data generated and analyzed for this study that are not included in this article and its Supporting Information are available from the authors upon reasonable request.

## Supporting Information Available

Figures S1–S16 and Tables S1–S5 (PDF)
Movie S1 (MP4)
Movie S2 (MP4)
