# Supplementary material for: Using Machine Learning to Understand the Causes of Quantum Decoherence in Solution-Phase Bond-Breaking Reactions
Source: J Phys Chem Lett. 2024 Jan 19;15(4):903–11. doi: 10.1021/acs.jpclett.3c03474 (PMC10839908; doi:10.1021/acs.jpclett.3c03474)
Supplement: Supplementary file 1 — jz3c03474_si_001.pdf [file jz3c03474_si_001.pdf]

Supplementary Information to "Using Machine Learning to Understand the Causes of Quantum Decoherence in Solution-Phase Bond Breaking Reactions"

Kenneth J. Mei, William R. Borrelli, Andy Vong, and Benjamin J. Schwartz*

Department of Chemistry & Biochemistry, University of California, Los Angeles, Los Angeles, CA 90095-1569 USA

E-mail: schwartz@chem.ucla.edu

Phone: (310) 206-4113

Contents

Simulation Details **S2**

Mixed Quantum/Classical and All-Classical Model S2

Simulation Setup S4

Additional Analysis **S5**

Difference in Local Solvent Potential S5

Local Solvent Density S6

Collective Solvent Velocity S6

Machine Learning Details **S7**

Balanced Random Forest Classification S7

Classifier Implementation S7

Feature Set & Training/Testing S8

SHAP Feature Importance S8

Principal Component Analysis (PCA) S8

Gaussian Process Regression S9

Regression Implementation S9

Feature Set & Training/Testing S9

Movies of Decoherence During Bond-Breaking **S19**

Simulation Details

Mixed Quantum/Classical Model

Simulation Details

Our mixed quantum/classical (MQC) molecular dynamics (MD) simulations consisted of two classical Na^+ cations, one fully quantum mechanical electron, and 1600 classical argon atoms in a 43.83 Å simulation cell. The size of the simulation cell was chosen to reproduce an appropriate solvent density at the simulation temperatures (1.26 g/mL at 120 ± 2 K) so that the system was well in the center of the liquid region of the L-J phase diagram. In all simulations, periodic boundary conditions were implemented with minimum image convention[1] and all interactions were tapered smoothly to zero at 16 Å over a 2 Å range with a center of mass-based switching function according to Steinhauser.[2] All simulations were performed in the microcanonical ensemble.

Because all interactions were taken to be pair-wise additive, the full Hamiltonian of the system is $\hat{H} = H^{\mathrm{cl}} + \hat{H}^{\mathrm{qm}}$. In atomic units, the classical portion of the Hamiltonian is given

by

$$H^{\mathrm{cl}} = \frac{1}{2} m_{\mathrm{Na}^+} \sum_{i=1}^{2} v_{\mathrm{Na}^+_i}^2 + \frac{1}{2} m_{\mathrm{solv}} \sum_{i=1}^{n_{\mathrm{solv}}} v_{\mathrm{solv}_i}^2 + U^{\mathrm{Na}^+-\mathrm{Na}^+}(|\boldsymbol{R}_{\mathrm{Na}^+_1} - \boldsymbol{R}_{\mathrm{Na}^+_2}|)$$
$$+ \sum_{i=1}^{n_{\mathrm{solv}}} \sum_{j>i}^{n_{\mathrm{solv}}} U^{\mathrm{solv}-\mathrm{solv}}(|\boldsymbol{R}_{\mathrm{solv}_i} - \boldsymbol{R}_{\mathrm{solv}_j}|) + \sum_{i=1}^{2} \sum_{j=1}^{n_{\mathrm{solv}}} U^{\mathrm{Na}^+-\mathrm{solv}}(|\boldsymbol{R}_{\mathrm{Na}^+_i} - \boldsymbol{R}_{\mathrm{solv}_j}|) \quad (1)$$

where v_{χ_i} is the velocity of the ith sodium ($\chi = \mathrm{Na}^+$) or solvent atom ($\chi = \mathrm{solv}$) at position $\boldsymbol{R}_{\chi_i}$ and mass m_χ. $U^{\chi-\gamma}$ is a classical potential between atom types χ and γ ($\chi, \gamma = \mathrm{Na}^+$ or solv).

The quantum Hamiltonian is given by

$$\hat{H}^{\mathrm{qm}} = \sum_{i=1}^{2} \frac{\hat{\boldsymbol{p}}_i^2}{2} + \sum_{i=1}^{2} \sum_{j=1}^{2} V^{\mathrm{Na}^+}(|\boldsymbol{R}_{\mathrm{Na}^+_j} - \hat{\boldsymbol{r}}_i|) + \sum_{i=1}^{2} \sum_{j=1}^{n_{\mathrm{solv}}} V^{\mathrm{solv}}(|\boldsymbol{R}_{\mathrm{solv}_j} - \hat{\boldsymbol{r}}_i|) \quad (2)$$

where $\hat{\boldsymbol{p}}_i$ and $\hat{\boldsymbol{r}}_i$ are the momentum and position operators for electron i, respectively, and V^χ is the pseudopotential representing the interaction between an electron and atom type χ.

The classical interaction between the two Na^+ cations was modeled through a point-charge Coulomb potential, $U^{\mathrm{Na}^+-\mathrm{Na}^+}(R) = 1/R$, since the short-range repulsion between the cores is negligible around the internuclear separation of Na_2^+.

Classical interactions were modeled with Lennard-Jones potentials:[1]

$$u_{ij}(r_{ij}) = \frac{1}{4\pi\epsilon_0} \frac{q_i q_j}{r_{ij}} + 4\epsilon_{ij} \left[\left(\frac{\sigma_{ij}}{r_{ij}} \right)^{12} - \left(\frac{\sigma_{ij}}{r_{ij}} \right)^{6} \right] \quad (3)$$

where r_{ij} is the distance between the ith and j^{th} solvent/Na^+ site, q_i is the charge on the ith site, ϵ_{ij} is the potential well depth, and σ_{ij} is the finite distance at which the inter-particle potential is zero. The Lennard-Jones parameters[3] used in this study are listed in Table S1.

Phillips-Kleinman (PK) pseudopotentials were used to account for the interactions be-

tween the classical particles and the quantum mechanical electron.[4] These PK potentials were modified with polarization potentials to correct for the frozen core approximation implicit in PK formalism.[5–7] For the e^--Ar interaction, V^{e-Ar}, we used a modified version of the pseudopotential developed by Gervais et al.,[8] described in detail in our previous work.[9] For the e^--Na^+ interaction, we used rigorously-derived psedopotentials previously developed by our group, the details of which can be found in Refs. 6 and 7, respectively. The final pseudopotential fits are presented here in Tables S2 and S3.

Simulation Setup

The eigenstates of the quantum mechanical valence bonding electron were expanded on a three dimensional grid. We used a grid that contained 32 × 32 × 32 grid points. These dimensions were chosen to keep the basis set as small as possible for each system while still capturing the spatial extent of the electronic wave function. We centered the grid in the middle of the simulation cell and shifted all classical particles relative to the grid every 500 fs to avoid leakage of the wave function off the edges of the grid. In this way, the wave function was always located roughly in the center of the simulation cell. The classical particles were shifted an integer number of grid spaces to avoid discontinuities in the quantum energy that would prevent total energy of the simulation from being conserved.[10] We used the velocity Verlet algorithm[1] to propagate the classical degrees of freedom ($\boldsymbol{v}_{\mathrm{Na^+}_i}$, $\boldsymbol{v}_{\mathrm{solv}_i}$, $\boldsymbol{R}_{\mathrm{Na^+}_i}$, and $\boldsymbol{R}_{\mathrm{solv}_i}$) of the Hamiltonian in Eqs. 1 and 2 in the microcanonical (N, V, E) ensemble. We determined the forces from the sum of the classical-classical and classical-quantum interactions described above. We used the implicit restart Lanczos method to iteratively solve the TISE for the ground state wavefunction at every 4 fs time step.[11] The quantum forces on the classical particles were then found using the Hellman-Feynman theorem:

$$\boldsymbol{F}_i^Q = -\langle\Psi|\,\nabla_{\boldsymbol{R}_i}\hat{H}\,|\Psi\rangle \tag{4}$$

where, $\boldsymbol{F}_i^Q$ is the quantum force on classical particle i at position $\boldsymbol{R}_i$. Because the wave function is expanded in a basis that does not functionally depend on the position of the classical particles, Eq. 4 is formally exact (in other words, there are no issues with Pulay forces from the basis functions changing with time).[12]

For this paper, we collected data from 210 non-equilibrium dissociation trajectories of Na_2^+ in liquid argon. Our initial configurations were generated from uncorrelated ground-state configurations and placed onto the electronic excited state at time zero of our non-equilibrium trajectories. The dynamics were propagated with nonadiabatic surface hopping, using Tully's fewest switches surface hopping (FSSH) algorithm.[13] For our analysis in the main text, all data is collected prior to any instances of surface hopping onto the electronic ground state and the decoherence of interest occurred adiabatically on the excited state before any surface hops took place in any of the trajectories.

Additional Analysis

Difference in Local Solvent Potential

The difference in local solvent potential measures the difference in integrated potential felt by the electron within a 2.6 radius around each Na^+. This is calculated using the value of the pseudopotential on our grid basis. We take all the grid points within a 2.6 radius of each Na^+ and sum the value of the potential at these points. The orange curve plotted in Figure 1 shows the absolute difference between the integrated pseudopotentials around each Na^+ in the 180-fs window prior to localization. Figure 1 shows that around the ~ 60 fs prior to localization, the value of the absolute difference in the integrated pseudopotential between each Na^+ sharply increases. The trend suggests that the local environments around each Na^+ become significantly different as the system approaches localization. The units of $|\Delta V^{e^- - Ar}|$ are expressed in the reduced units of the pseudopotential, where A = 0.28 Hartrees.

Local Solvent Density

The local density around each Na^+ is calculated using a density field method from the work of Willard and Chandler,[14] with a coarse-graining length of 1.7 . The grid used in the calculation is the electron grid basis. The curves shown in Figure 2 are the average density values of grid points within a 2.0 radius of each Na^+. At ∼60 fs prior to localization, there is clearly a crossover between the local solvent density around each Na^+, where the Na^+ that receives the electron experiences an increase in local solvent density and the other Na^+ sees the opposite trend. The opposing directions of solvent density on each Na^+ correlate well with a ∼60 fs localization event and the idea of out-of-phase solute-solvent collisions driving localization.

Collective Solvent Velocity

To visualize the solute-solvent collisions coinciding with decoherence we calculated the components of the collective solvent velocities moving away or towards each Na^+. We weight the distance of each solvent atom to each grid point using the same weighting function as that in the density field. To account for the motions of the Na^+ we subtract off its velocity vector from all argon velocity vectors to view the velocity field in a locally stationary frame. We then sum the distance weighted instantaneous relative velocity vectors to measure the collective argon velocities around each grid point. For instance, two argons at the same distance from a grid point with exactly opposite instantaneous velocities will return a collective velocity of zero at that grid point.

In order to measure net direction of collective solvent motion (ϕ), the grid is generated as a spherical shell around each Na^+ with a radius of 2.8 . The relative solvent velocity field is calculated on this grid and we take the component of the collective velocity along a normal vector to the grid surface. A visualization of the spherical grid with the normal components of the collective velocity vectors are shown in Figure 3 panel c(localized) and panel d(unlocalized) for a single timestep during localization. The net direction of the

collective solvent velocities is then the sum of all normal vectors on the shown spherical surface. The plots in Figure 3 panel a and panel b are the ensemble averaged net collective solvent velocities in the specified time regimes.

In Figure 3 panel a, the net collective solvent velocity is negative in the first 100 fs after excitation corresponding to the inward motion of solvent velocity as the fragments dissociate. After the caging event which occurs at ∼100 fs after excitation for this system, we observe a positive value for ϕ, indicating the net motion of solvent away from each Na^+. In this time regime, the two Na^+ atoms do not experience significant differences in their local solvent motions and suggest that the caging collisions with the two fragments occur simultaneously, on average. When viewed as the time to localization, ϕ shows significant differences between the localized and unlocalized Na^+. Most notably, ϕ is negative for the 60 fs leading up to localization for the Na^+ that receives the electron, showing the net inward motion of solvent in this time regime. As expected, the opposing Na^+ has a positive value for ϕ, suggesting a lack of collisions is needed for localization.

Machine Learning Details

Balanced Random Forest Classification

Classifier Implementation

Our Balanced Random Forest (BRF) classifier was implemented in Python version 3.9.4 using imblearn version 0.7.0 along with scikit-learn version 0.24.2. Table 3 shows the features used to train the model, along with a feature description. Hyper-parameter optimization was done using a grid-search method to tune n_estimators and the max_depth of the random forest model. The chosen hyper-parameters are shown in Table 4.

Features that were not used in the final model but were included in the initial feature set included atom-centered-symmetry functions (ACSFs), spherical pseudopotential values,

and effective volume values for each sodium. Spherical pseudopotential values were identical to the integrated pseudopotential, except that the values were integrated on the surface of a sphere around each sodium rather than the entire volume of the sphere. ACSFs were implemented following the formalism of Behler[15] and hyperparameters were tuned using a random search method.

Feature Set & Training/Testing

Since every 9-tuple of our data set was taken from a single MD trajectory, we split our data into train and test splits by allocating certain trajectories for training and testing. This eliminates the possibility of data leakage where correlated examples from the same trajectory end up in both the training and testing data, potentially leading to inflated model performance. We also trained models using a random test/train split methodology and nearly identical results were obtained.

The learning curve for the BRF classifier is shown in Figure 5. Table 5 shows the balanced accuracy scores for the various ways we evaluated model performance. We also tested model performance on a curated test set of examples that are near localization (absolute value charge difference near but not exceeding 0.90) and achieved a balanced accuracy score of 0.685.

Figures 6 and 7 shows the standardized distributions of each feature for the training and test sets, respectively.

SHAP Feature Importance

Figures 8-10 show the SHAP feature importance charts for each class.

Principal Component Analysis (PCA)

We additionally did exploratory principal component analysis on the classifier data set. This is shown in Figures 11-14.

Gaussian Process Regression

Regression Implementation

Our Gaussian Process (GP) regression model was implemented in Python version 3.9.4 using scikit-learn version 0.24.2. A radial basis function (squared-exponential) kernel was used, with an initial length scale setting of 1.0, and a minimum and maximum length scale of 1E-2 and 1E2 respectively.

Feature Set & Training/Testing

Our feature space for regression included several physically motivated encodings of the solute local environment. Atom-centered symmetry functions (ACSFs) corresponding to equations 5 and 6 with the cutoff function given by equation 7 were computed using an in-house Mathematica code implemented in Mathematica 13.1. For all ACSFs, R_{ij} and R_{ik} denote sodium-argon distances, R_{ik} denotes argon-argon distances, and θ_{ijk} corresponds to argon-sodium-argon angles. The hyper-parameters η, $_S$, λ, and ζ were optimized using a random search method.

$$G_{radial,i} = \sum_j e^{-\eta(R_{ij}-R_s)^2} \cdot f_c(R_{ij}) \tag{5}$$

$$G_{angular,i} = 2^{(1-\zeta)} \sum_j \sum_{k \neq j} (1 + \lambda cos\theta_{ijk})^{\zeta} \cdot e^{-\eta(R_{ij}^2+R_{ik}^2+R_{jk}^2)} \cdot f_c(R_{ij}) \cdot f_c(R_{ik}) \cdot f_c(R_{jk}) \tag{6}$$

$$f_{cut}(R_{ij}) = \begin{cases} 0.5[cos(\frac{\pi R_{ij}}{R_c}) + 1] & \text{if } R_{ij} \leq R_c \\ 0 & \text{if } R_{ij} > R_c \end{cases} \tag{7}$$

In addition to the ACSFs for each sodium atom, we included dimer bond distance, spherical sodium-argon pseudopotential, and effective volumes around each sodium, to our feature

space. Note that the spherical pseudopotential differs from the integrated pseudopotential. The spherical pseudopotential feature was integrated over the surface of a 2.8 Åsphere centered at the sodium core, while the integrated pseudopotential feature was integrated inside a 2.8 Åsphere centered at the sodium core.

Figures 15 and 16 shows the normalized distributions of each feature for the training and test set respectively.

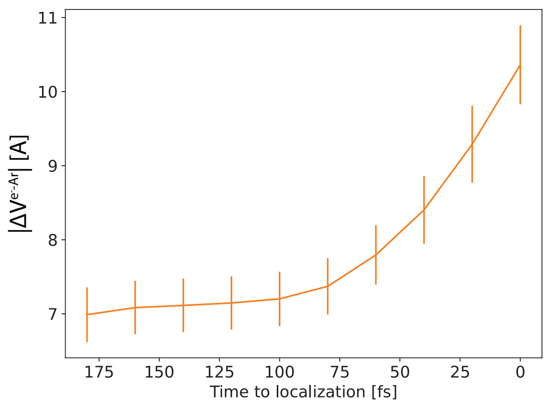

Figure 1: Ensemble averaged absolute difference in local solvent potential felt by the electron between each Na^+, 180fs before decoherence.

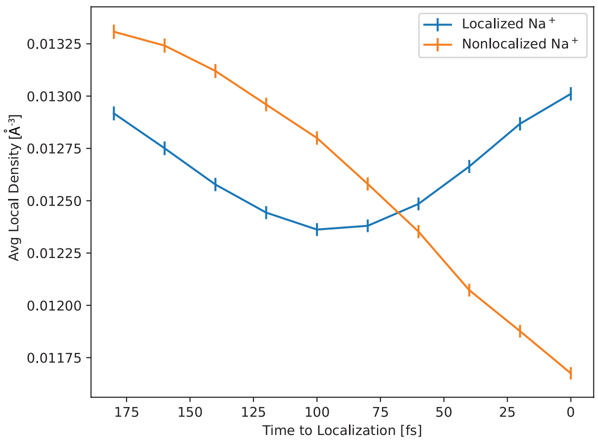

Figure 2: Ensemble averaged local solvent density around each Na^+. Blue curve corresponds to the Na^+ that receives the electron and orange curve corresponds to the opposing Na^+

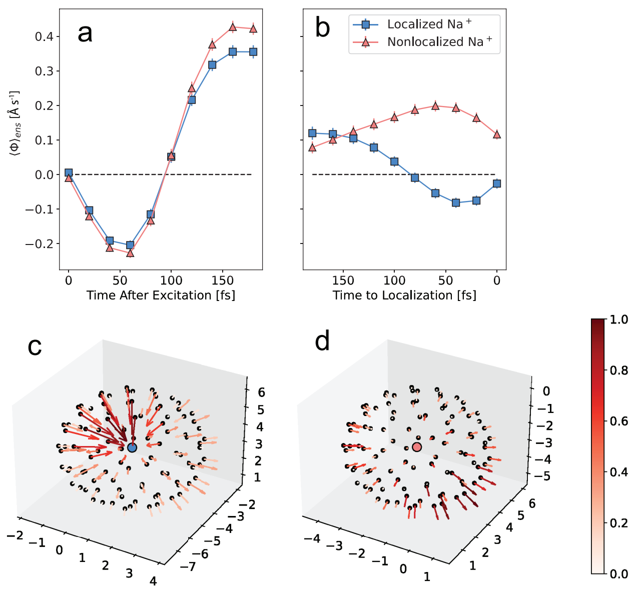

Figure 3: Net direction of collective solvent motion for each Na^+, blue for localized Na^+ and pink for unlocalized Na^+. Panel a shows the collective solvent velocity in the first 180 fs showing the caging event. Panel b shows the 180fs before decoherence in each trajectory, exhibiting a difference in the net direction of solvent motion between each fragment. Negative values correspond to net solvent motion inward and positive values correspond to outward solvent motion. Panel c and d visualize a representative snapshot of the collective solvent motion for the localized and unlocalized Na^+, respectively.

Table 1: **Lennard-Jones and Coulomb Potential Parameters for the Solute-Solvent Systems Studied in This Work.**

	σ (Å)	ϵ (kJ/mol)	q (e)
Argon	3.405	0.996	0.0
Na^+	2.69	0.5144	+1.0

Table 2: **Parameters used in the construction of the sodium-electron pseudopotential, $\phi(r) = \sum_{i=1}^{3} c_i e^{-\alpha_i r^2}$.** Further details of the sodium-electron pseupotential can be found in Ref 6.

i	c_i (a.u.)	α_i (a.u.)
1	-16.3145	0.124293
2	0.0455219	0.0322129
3	16.3213 81	0.124181

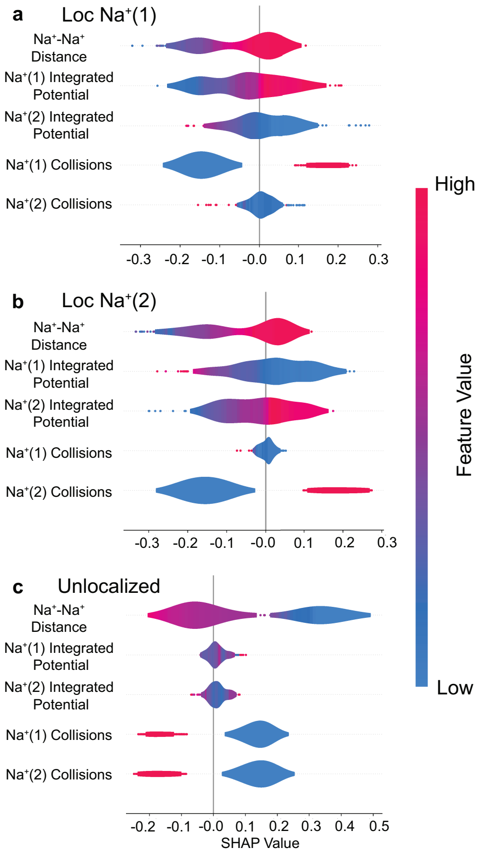

Figure 4: Full SHAP analysis for the BRF classifier model

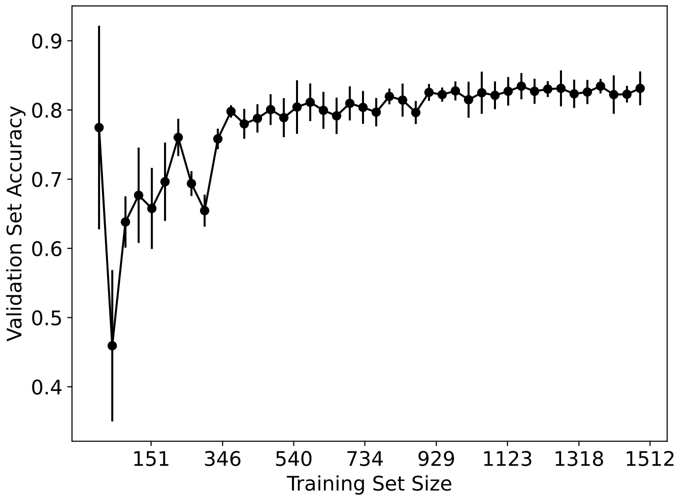

Figure 5: Learning curve for BRF classifier model trained on 151 to 1,512 training data points and tested on 378 data points using a single train/test split.

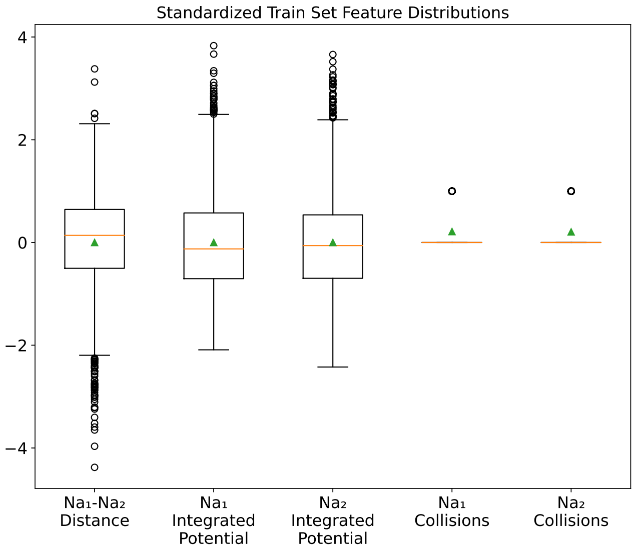

Figure 6: Distributions of the standardized features of the training set for the balanced random forest model.

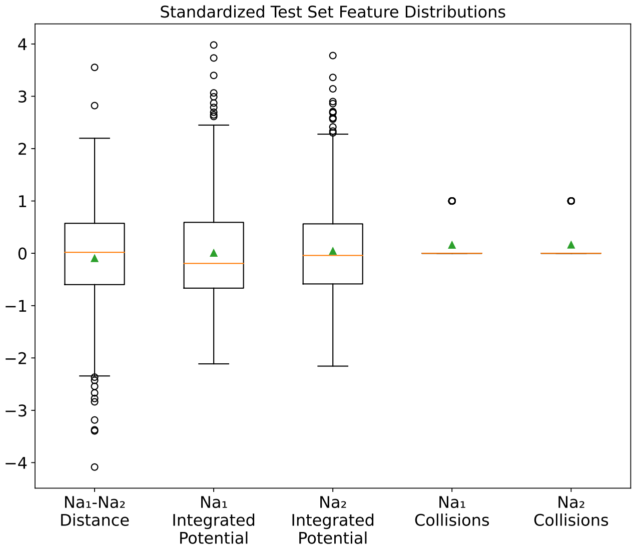

Figure 7: Distributions of the standardized features of the test set for the balanced random forest model.

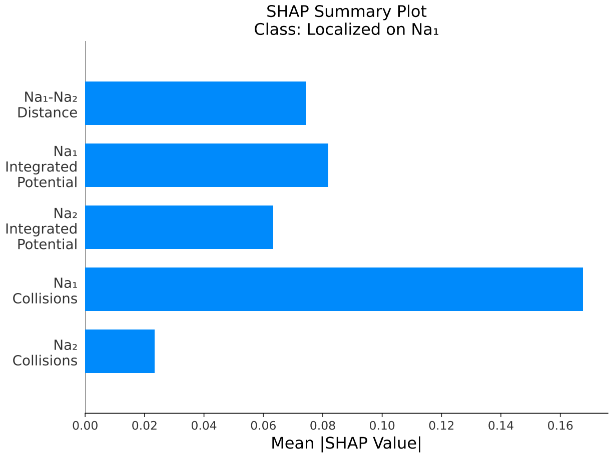

Figure 8: SHAP feature importance for class 1 - localized on Na_1

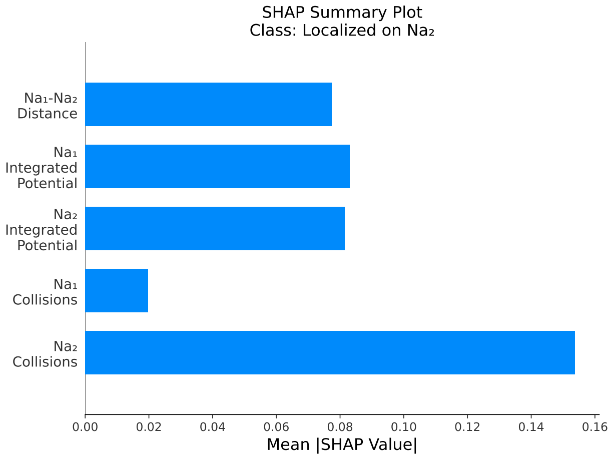

Figure 9: SHAP feature importance for class 2 - localized on Na_2

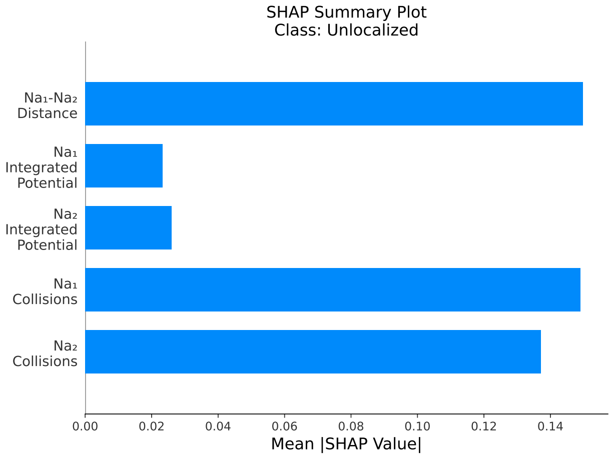

Figure 10: SHAP feature importance for class 3 - unlocalized

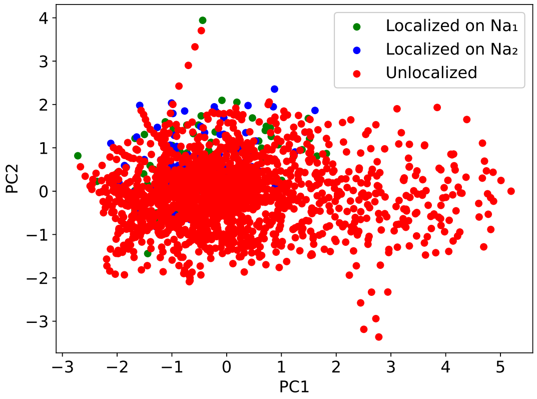

Figure 11: Plot of principal components 1 and 2 for the full feature set.

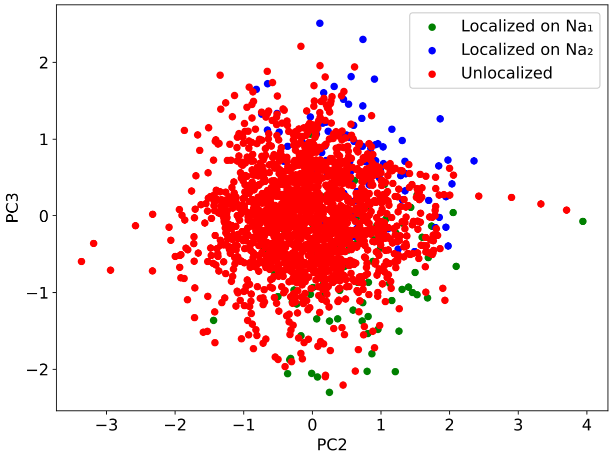

Figure 12: Plot of principal components 2 and 3 for the full feature set.

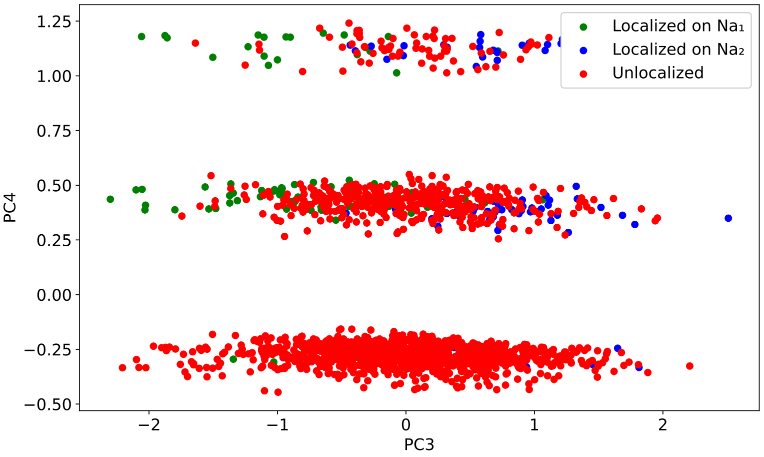

Figure 13: Plot of principal components 3 and 4 for the full feature set.

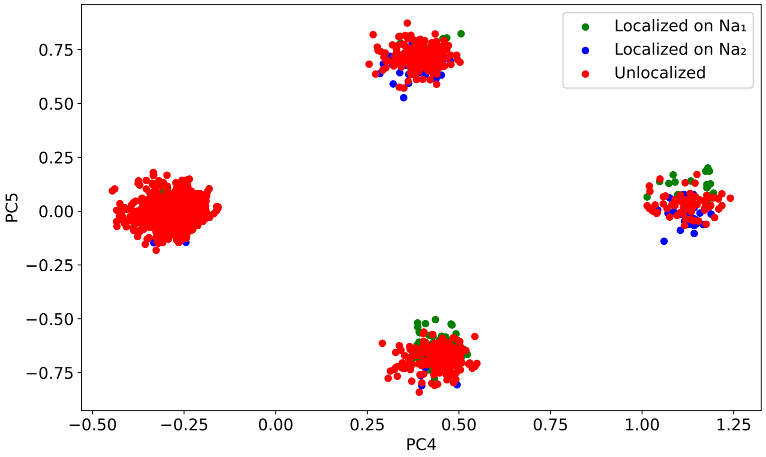

Figure 14: Plot of principal components 4 and 5 for the full feature set.

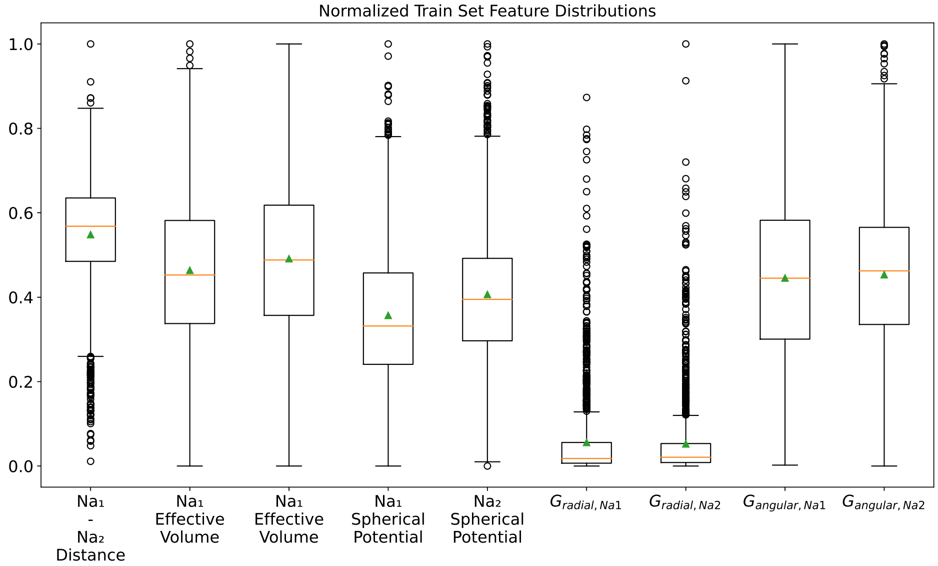

Figure 15: Distributions of the normalized features of the training set for the balanced random forest model.

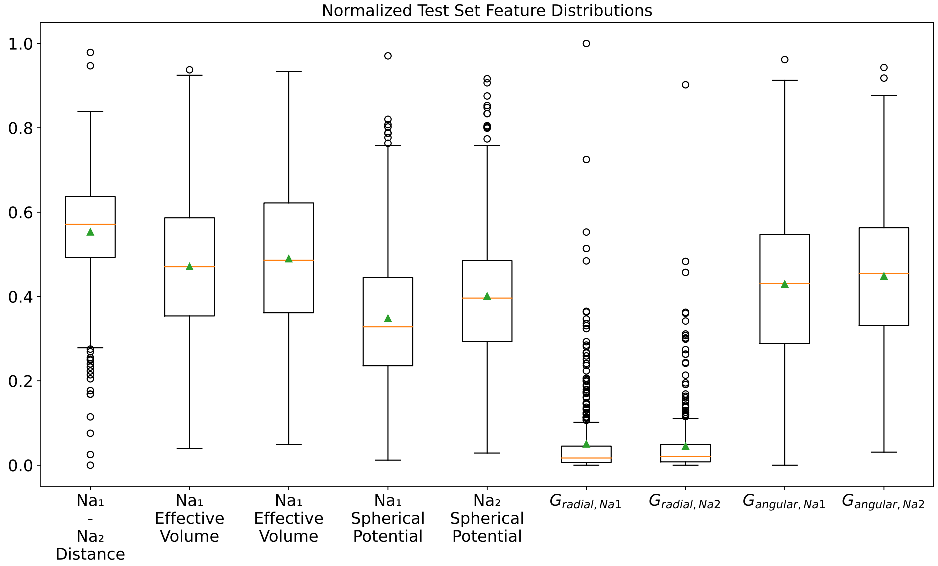

Figure 16: Distributions of the normalized features of the test set for the balanced random forest model.

Table 3: **Machine learning features for the BRF classifier model along with feature descriptions**

Feature Name	Description
bond length	Distance between Na^+ (1) core and Na^+ (2) core in the dimer molecule
integrated pseudopotential Na^+ (1)	Na-Ar pseudopotential integrated over a 2.8 angstrom sphere centered on the Na^+ (1) core
integrated pseudopotential Na^+ (2)	Na-Ar pseudopotential integrated over a 2.8 angstrom sphere centered on the Na^+ (2) core
Na^+ (1) binary collision vector	Binary collision encoding for Na^+ (1). Value of 1 for collision, 0 for no collision.
Na^+ (2) binary collision vector	Binary collision encoding for Na^+ (1). Value of 1 for collision, 0 for no collision.

Table 4: **Chosen hyper-parameters for the Balanced Random Forest classifier model, evaluated using a grid-search method.**

Hyper-Parameter	Best Value
n_estimators	64
max_depth	5

Table 5: **Balanced accuracy scores over the various model validation methodologies for the BRF classifier. A single train/test split, cross-validation, and replicate train/test splits produced very similar balanced accuracy scores for the BRF model. This indicates that the reported balanced accuracy score is robust to sampling biases. Note that the error shown in standard error of the mean.**

Method	Balanced Accuracy Score
Single Train/Test Split	0.799
K=5 Cross Validation	0.789 $\pm$0.0132
100 Replicate Train/Test Split	0.785 $\pm$0.00376

Movies of Photoinduced Bond-Breaking

Attached to the SI are movies of the photoinduced bond breaking of Na_2^+ in the gas phase as well as in liquid Ar. The blue spheres represent the Na^+ cores and the isosurface represents the charge density of the bonding electron. Pink spheres represent the Ar atoms of the bath. From the gas phase bond breaking we see that the bonding electron remains in a superposition of positional states centered on each Na^+ for the duration of the bond breaking process as there is no environment to break the symmetry and collapse its wavefunction. In the condensed-phase dissociation we see the bonding electron collapse onto one of the Na^+ nuclei due to the fluctuations in the solvent environment.

Table 6: **Machine learning features for the GP regression model along with feature descriptions**

Feature Name	Description
bond length	Distance between Na^+ (1) core and Na^+ (2) core in the dimer molecule
spherical pseudopotential Na^+ (1)	Na-Ar pseudopotential integrated over the surface of a 2.8 angstrom sphere centered on the Na^+ (1) core
spherical pseudopotential Na^+ (2)	Na-Ar pseudopotential integrated over the surface of a 2.8 angstrom sphere centered on the Na^+ (2) core
effective volume Na^+ (1)	Free volume around the Na^+ (1) core
effective volume Na^+ (2)	Free volume around the Na^+ (2) core
$G_{radial,Na^+(1)}$	G_{radial} evaluated from the Na^+ (1) core on the nearest 50 argon atoms
$G_{radial,Na^+(2)}$	G_{radial} evaluated from the Na^+ (2) core on the nearest 50 argon atoms
$G_{angular,Na^+(1)}$	$G_{angular}$ evaluated from the Na^+ (1) core on the nearest 50 argon atoms
$G_{angular,Na^+(2)}$	$G_{angular}$ evaluated from the Na^+ (2) core on the nearest 50 argon atoms

References

(1) Allen, M. P.; Tildesley, D. J. *Computer Simulation of Liquids*; Oxford University Press; Oxford, 1992.

(2) Steinhauser, O. Reaction Field Simulation of Water. *Mol. Phys.* **1982**, *45*, 335–348.

(3) Balbuena, P. B.; Johnston, K. P.; Rossky, P. J. Molecular Dynamics Simulation of Electrolyte Solutions in Ambient and Supercritical Water. 1. Ion Solvation Free Energies of Solvation for Cl. *J. Phys. Chem.* **1995**,

(4) Phillips, J. C.; Kleinman, L. New Method for Calculating Wave Functions in Crystals and Molecules. *Phys. Rev.* **1959**, *116*, 287–294.

(5) Larsen, R. E.; Glover, W. J.; Schwartz, B. J. Does the Hydrated Electron Occupy a Cavity? *Science* **2010**, *329*, 65–69.

(6) Smallwood, C. J.; Larsen, R. E.; Glover, W. G.; Schwartz, B. J. A Computationally Efficient Exact Pseudopotential Method. I. Analytic Reformulation of the Philips-Kleinman Theory. *J. Chem. Phys.* **2006**, *125*, 074102.

(7) Smallwood, C. J.; Mejia, C. N.; Glover, W. J.; Larsen, R. E.; Schwartz, B. J. A Computationally-Efficient Exact Pseudopotential Method. II. Application to the Molecular Pseudopotential of an Excess Electron Interacting with Tetrahydrofuran (THF). *J. Chem. Phys.* **2006**,

(8) Gervais, B.; Giglio, E.; Jacquet, E.; Ipatov, A.; Reinhard, P. G.; Suraud, E. Simple DFT Model of Clusters Embedded in Rare Gas Matrix: Trapping Sites and Spectroscopic Properties of Na Embedded in Ar. *J. Chem. Phys.* **2004**, *121*, 8466–8480.

(9) Glover, W. J.; Schwartz, B. J. How Does a Solvent Affect Chemical Bonds? Mixed Quantum/Classical Simulations with a Full CI Treatment of the Bonding Electrons. *J. Phys. Chem. Lett.* **2010**, *1*, 165–169.

(10) Glover, W. J.; Larsen, R. E.; Schwartz, B. J. The Roles of Electronic Exchange and Correlation in Charge-Transfer-to-Solvent Dynamics: Many-Electron Non-Adiabatic Mixed Quantum/Classical Simulations of Photoexcited Sodium Anions in the Condensed Phase. *J. Chem. Phys.* **2008**, *129*, 164505.

(11) Lehoucq, R. B.; Sorensen, D. C.; Yang, C. *ARPACK Users' Guide*; Society for Industrial and Applied Mathematics: Philadelphia, 1998.

(12) Glover, W. J.; Larsen, R. E.; Schwartz, B. J. First Principles Multi-Electron Mixed Quantum/Classical Simulations in the Condensed Phase. I. An Efficient Fourier-Grid Method for Solving the Many-Electron Problem. *J. Chem. Phys.* **2010**, *132*, 1–11.

(13) Tully, J. C. Molecular Dynamics with Electronic Transitions. *J. Chem. Phys.* **1990**,

(14) Willard, A. P.; Chandler, D. Instantaneous Liquid Interfaces. *J. Phys. Chem. B* **2010**, *114*, 1954–1958.

(15) Behler, J. Atom-centered Symmetry Functions for Constructing High-Dimensional Neural Network Potentials. *J. Chem. Phys.* **2011**, *134*, 074106.
